# Spontaneous primary pneumomediastinum: is it always benign?

**DOI:** 10.1186/s13256-021-02701-z

**Published:** 2021-03-25

**Authors:** Berhanu N. Alemu, Ephraim T. Yeheyis, Abraham G. Tiruneh

**Affiliations:** Cardiothoracic Unit, Department of Surgery, School of Medicine, College of Health Sciences, Addis Ababa University, Addis Ababa, Ethiopia

**Keywords:** Spontaneous pneumomediastinum, Mediastinal emphysema, Pneumorrhachis

## Abstract

**Background:**

Spontaneous Pneumomediastinum is a rare disease. It could be a simple and self-limited condition or be a life-threatening complication of underlying diseases. The therapeutic options also differ by the cause. This systematic review was done to provide, as far as we know, the first attempt to broadly assess the clinical feature, predisposing factors, possible management, and outcome of spontaneous primary pneumomediastinum.

**Methods:**

In addition to the two patients treated at our hospital, a Pub Med Search for literature on case reports of spontaneous pneumomediastinum published in English up to November 2018 was done. We extracted data on patients' demographic characteristics, symptoms, timing, diagnosis, management, and outcome of the treatment were analyzed based on the preferred Reporting Items for Systematic reviews and Meta-analysis (PRISMA)

**Result:**

A total of 339 cases were reviewed. 71.7% of them were male. The Mean age affected was 22.4 ± 11.3 years. Chest pain, 196 (57.8%), is the most common presenting symptom, followed by dyspnea, 156 (46%), cough 95 (28%), neck swelling 92 (27.13%), cervical pain 88 (25.9%), dysphagia 39 (11.5%), odynophagia 37 (10.9%), and Dysphonia 14 (4.1%). Fifty-seven patients (16.8%) had a prior history of Asthma, 19 (5.6%) had Connective Tissue Disorders, and 12 (3.5%) had associated malignancy as an identified risk factor. In 35 (10.3%) patients, spontaneous pneumomediastinum was found incidentally. The mean number of days before the clinical resolution of spontaneous pneumomediastinum was 6.65 ± 11.8 days and the average hospital stay was 4.15 ± 1.93 days. Nineteen (5.6%) patients have died as a result of the underlying disease not related to SPM.

**Conclusion:**

Spontaneous pneumomediastinum is uncommon, usually benign, a self-limited disorder that commonly occurs in a young adult without any apparent precipitating factor or disease. Spontaneous pneumomediastinum usually responds very well to conservative treatment without recurrence. However, secondary causes should be ruled out to minimize the unfavorable outcome.

## Introduction

Spontaneous pneumomediastinum (SPM) is a rare usually benign condition, characterized by the presence of air in the mediastinum. It is usually associated with subcutaneous emphysema and occasionally with pneumothorax. Rarely, it may associate with pneumorrhachis (air within the spinal epidural space) [1].

SPM is uncommon in adults; it primarily affects young male individuals with the male to female ratio of 8:1 [2, 3]. Different kinds of literature reported that, the number of cases per hospital admission range from 1 in 800 to 1 in 42,000. Of these cases, approximately 1% have a history of asthma [3]. Other risk factors identified are vomiting, inhaled drug use, smoking, and the Valsalva maneuver [4].

The main associated symptoms of SPM are chest pain, dyspnea, coughing, dysphonia, dysphagia, and cervical pain. The diagnosis is established by imaging techniques, especially chest x-ray and chest Computed tomography scan (CT-scan). It is usually self-limited, which justifies only symptomatic relief [5, 6].

The current study was performed to summarize the available data on different aspects of SPM, which includes the cause, clinical presentation, diagnostics and treatment modalities, and any possible complications identified. In addition to the two cases recently encountered at our hospital, 339 published cases that were reported by different articles with sufficient data on the patients' clinical presentation, disease course, and management outcome were reviewed and analyzed.

## Case reports

### Case 1

A 25-year black male patient presented to our emergency department with complaints of shortness of breath (SOB) and diffuses body swelling over four days. The swelling initially started at the neck but later involved the chest and the whole body. He also had a stridor and dry cough. He was diagnosed to have Asthma four months before the presentation and was put on salbutamol puff but there was no significant improvement.

On presentation, he was acutely sick looking in respiratory distress. His vital signs were blood pressure: 110/90 mmHg, pulse rate: 100/min, respiratory rate: 30/min, SpO_2_ 98% on 3 L/min of intranasal oxygen, temperature: 36.60c. He had a puffy face with right side peri-orbital swelling. His neck was visibly swollen. Diffuse subcutaneous emphysema all over the chest, the abdomen, and even reached to lower extremities. Chest X-ray showed Pneumomediastinum with extensive subcutaneous emphysema.

With the possible diagnosis of tracheal injury, the patient was taken to the operation room and under general anesthesia neck exploration was done. But there was no visible bubbling and no visible tracheal injury appreciated. The wound was closed and the patient was transferred to intensive care unit (ICU). After four days of staying in the ICU and was being managed with ceftriaxone + metronidazole, & hydrocortisone. After a total of 7 days of stay in our hospital, he was discharged improved.

**Fig. 1.**
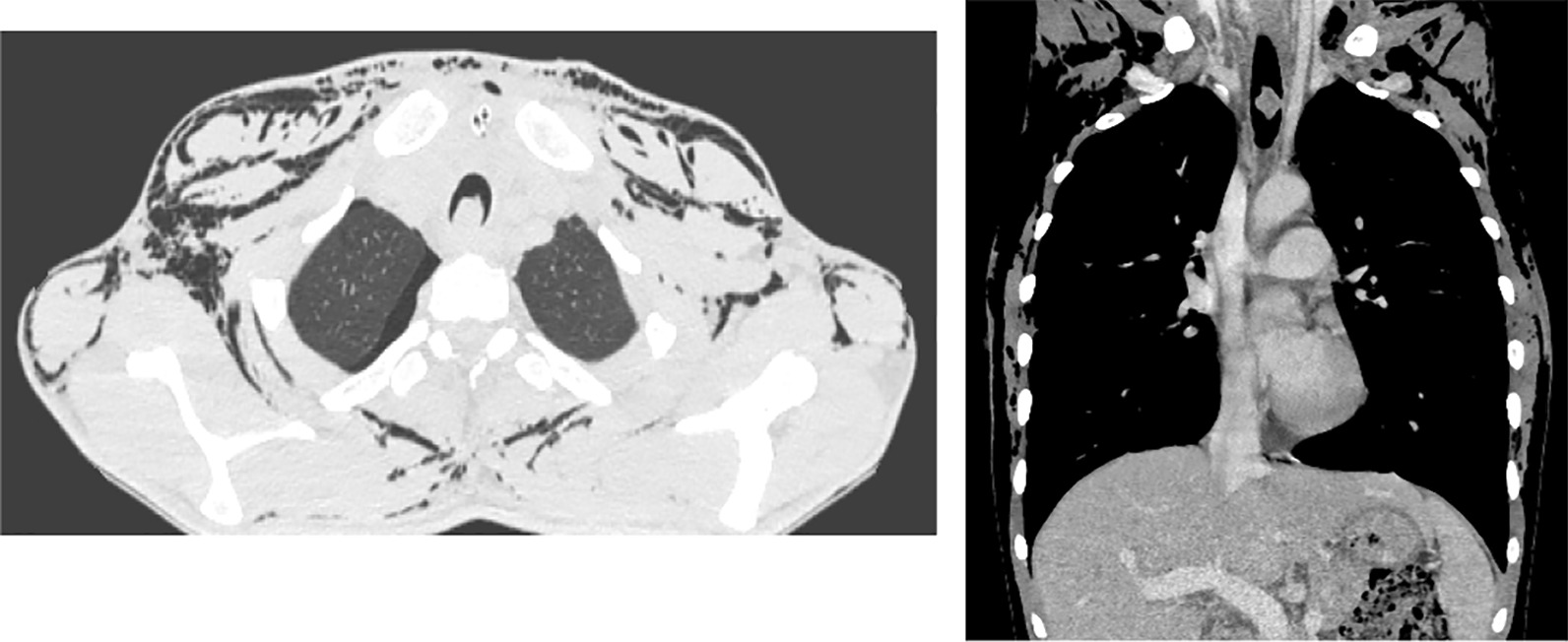
Computed tomography Scan that showed extensive subcutaneous emphysema, intraluminal soft tissue mass arising from the right posterolateral aspect of trachea about 5 cm from the carina with small air collection in the anterior mediastinum, right pleural, and anterior pericardial spaces

A week later, he came again to the emergency room with similar complaints but higher intensity. CT Scan was done and showed extensive subcutaneous emphysema, with air collection within the anterior mediastinum, right pleural, and anterior pericardial spaces. Additionally, an intraluminal soft tissue mass arising from the right posterolateral aspect of the trachea was found (Fig. 1).

With the diagnosis of tracheal mass, SPM, and pneumothorax, the patient was taken to the operating room for surgery. After general anesthesia, using single-lumen tracheal intubation, the chest was opened through a median sternotomy. The trachea was dissected circumferentially at the level of thoracic inlet and trachea opened and the tumor was found around the 4th tracheal ring for which tracheal resection and anastomosis was done.

**Fig. 2.**
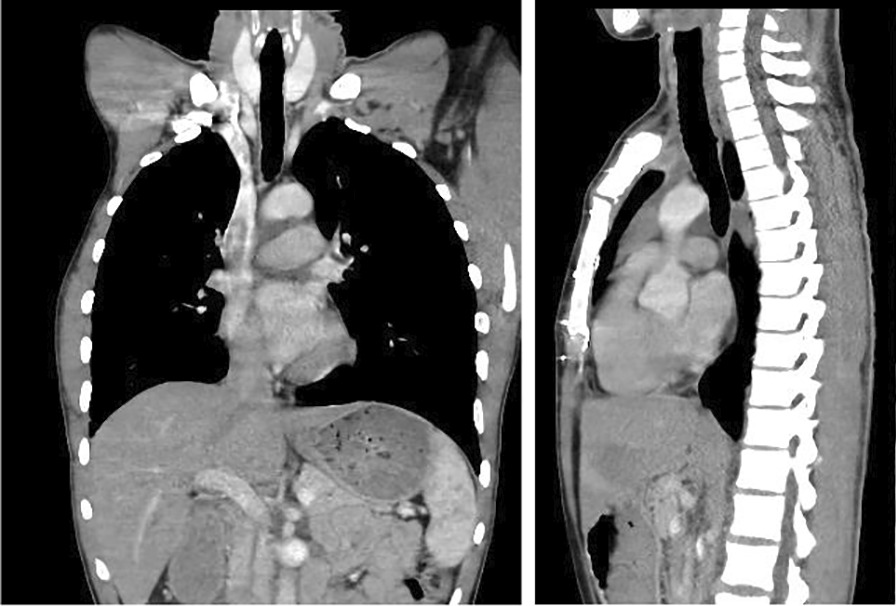
Unremarkable Chest computed tomography scan done after two years following tracheal resection

Postoperatively he had significant improvement with the SOB, subcutaneous emphysema, and pneumothorax. He was discharged on the 11th post-op day. The pathology exam of the tracheal mass was reported to be Low-grade soft tissue sarcoma. The patient was followed for three years and has no recurrence. CT-scan also showed a normal result (Fig. 2).

### Case 2

A 23-year-old black male presented to the adult emergency department in Tikur Anbessa Hospital after being referred from Zeweditu Memorial Hospital with the diagnosis of subcutaneous emphysema 20 to Pneumomediastinum. He initially presented with 03 days history of Neck and upper chest swelling which was preceded by upper respiratory tract infection and flu-like symptoms, cough, rhinorrhea, and low-grade fever. He also had pain during swallowing. He is a known asthmatic patient for the past year on salbutamol puff. Otherwise, he has no history of chest pain, hoarseness of voice, vomiting, diarrhea, dizziness, palpitations, change in exercise tolerance, or diaphoresis. He doesn't have a history of drug inhalation, constipation, prior admission or intervention, allergy history, or a history of smoking.

On physical examination, he is acutely sick looking with a grossly swollen face and neck but he is not short of breath and was non-toxic. The temperature of 36.8 °C, blood pressure of 100/70 mm Hg, respiratory rate of 20, pulse rate of 72 and maintains oxygen saturation at 94% with room air. Neck examination revealed gross swelling with subcutaneous crepitation anteriorly, laterally, and posteriorly including the upper chest. The trachea is central, no jugular vein distention and no audible stridor. Chest examination only revealed diffused wheezing all over the lung field aside from the upper chest subcutaneous crepitation. Otherwise, the examination of other systems was unremarkable.

He was investigated with complete blood count, urinalysis, chest x-ray, Echocardiography, and CT-scan of the chest. ECHO revealed a small pneumopericardium otherwise normal left ventricular systolic and diastolic function. Chest CT showed Pneumomediastinum with subcutaneous emphysema on the anterior and lateral neck and chest wall. The upper GI endoscopy was normal. With the assessment of Spontaneous Pneumomediastinum, the patient was put on intranasal oxygen, maintenance fluid, and kept NPO. Salbutamol puff and Intravenous dexamethasone QID was also given. He has shown marked improvement with this conservative treatment and was discharged from the hospital after 3 days.

## Materials and method

### Data sources

In addition to the above two case reports, we searched the database of PubMed up to November 2018. Whenever the search engine suggests a link to a related article, it was also explored.

### Search term

The main search term used was "Spontaneous Pneumomediastinum". Subsequently, additional keywords like "mediastinal emphysema", "subcutaneous emphysema", "secondary Pneumomediastinum" were included.

### Identification of articles

All Full-text English articles that mention SPM were included in the study without any restriction of age, sex, cause, place, or year of publication. Reports were excluded from further analysis if they are duplicates, did not clearly describe diagnostics and treatment of SPM.

The initial database's search has yielded a total of 110 articles that fulfill the inclusion criteria. Of these, 13 articles were excluded (1 review of an article, 4 retrospective analysis, 5 case series, 2 communications and 1 article that only describes the radiological finding without adequate clinical data). Finally, a total of 97 articles (including the two cases reported above) were found eligible for analysis.

### Data extraction and analysis

Data were extracted from each case reports using a standardized data extraction form by two reviewers. All studies identified were case reports. The patients' demographic characteristics, symptoms, timing, methods of diagnosis, management, and outcome of treatment were analyzed.

## Results

**Fig. 3:**
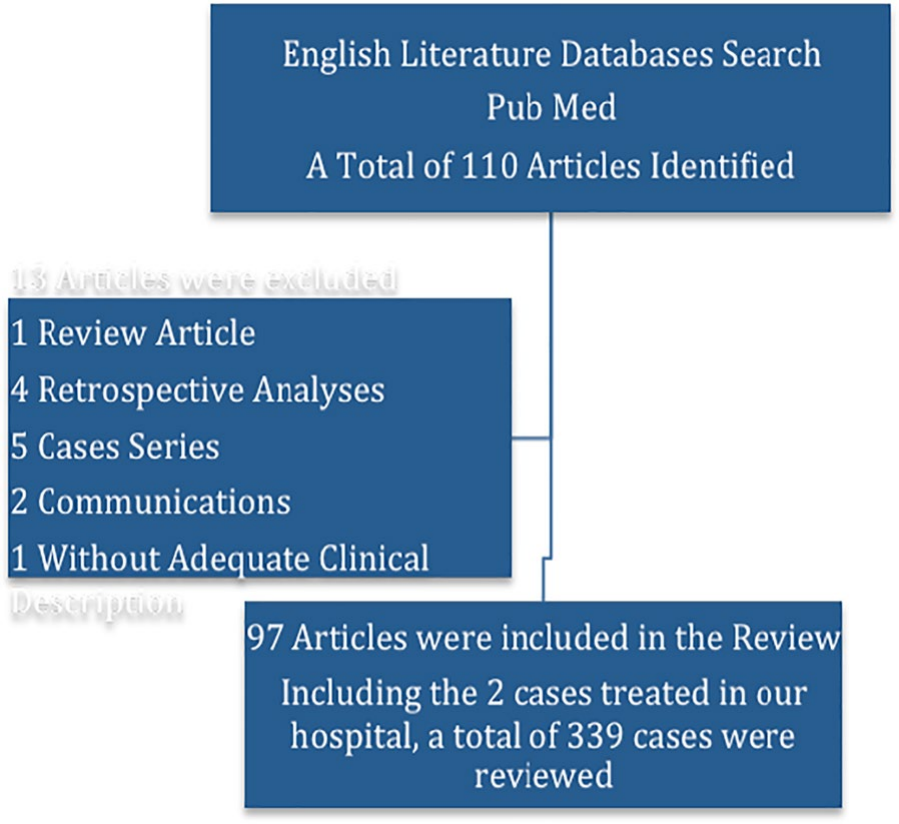
Flow Chart of the Review article Selection (*some papers have 2 or 3 cases reported on a single article)

A total of 339 cases of SPM were reviewed. The 337 cases from the literature and the 2 cases reported before are included in the analysis. Until 1960 there were only 4 (1.2%) cases reported, by the year 2000, the number of literature published has reached 76 (22.4%). Since then, 259 (76.4%) cases with SPM were published. SPM was more frequently reported from Asia (176 [51.9%]) followed by USA (95 [28%]), Europe (53 [15.6%]), Canada (6 [1.8%]), South America (4 [1.2%]), Africa (3[0.88%]), and Australia (2 [0.5%]) (Fig. 3).

### Clinical features

The patients' age ranged from neonate to 88 years (mean 22.4 ± 11.3 years), and the median age during the time of the diagnosis was 21 years. Out of the 339 patients, 243 (71.7%) were men and 96 (28.3%) were females (Table 1).

**Table 1:** Demographic characteristics, presenting symptoms and signs of 339 patients with Spontaneous Pneumomediastinum

*Demographics*	Frequency
Age (years)	22.4 ± 11.3
Sex	
Male	243 (71.7)
Female	96 (28.3)
Hospital Stay (Days)	4.15 ± 1.93
*Symptoms*	
Dyspnea	156 (46)
Chest pain	196 (57.8)
Cough	95 (28)
Neck swelling	92 (27.13)
Cervical Pain	88 (25.9)
Dysphagia	39 (11.5)
Odynophagia	37 (10.9)
Dysphonia	14 (4.1)
*Signs*	
Crepitus over Chest and Neck	154 (45.4)
Tachypnea	21 (6.1)
Fever	16 (4.7)
Hamman’s sign	38 (11.2)

Chest pain is the most common presenting symptom seen in 196 (57.8%) patients and it is followed by dyspnea (156 [46%]). Other symptoms identified in descending order were Cough 95 (28%), neck swelling 92 (27.13%), cervical pain 88 (25.9%), dysphagia 39 (11.5%), odynophagia 37 (10.9%), and Dysphonia 14 (4.1%). In about 35 (10.3%) cases, SPM was incidentally found during an investigation done for other problems with no identifiable symptom. Looking into the presence of other associated symptoms, 214 (63.1%) cases had no associated symptoms were as pleuritic chest pain was seen in 39 (11.5), fever in 16 (4.7%), easy fatigability in 7 (2.06%), and Numbness of legs in 3 (0.88%). Ninety-eight case reports have specified the duration of symptoms before seeking medical advice. Of them, 46 (46.9%) of cases present to the hospital within a day of onset of symptoms. The rest seek medical advice in 2 days, 23 (23.4%), 2-7 days, 21 (21.4%), >7 days, 8 (8.2%), and not specified in 301 (75.4%) cases.

Physical examination was not remarkable in 103 (30.4%) case reports. The rest of cases had finding like Crepitus over Chest and Neck, (154 (45.4%)), Crepitus over the Neck, (25 (7.3%)), Crepitus over the Shoulder and Neck, (21 (6.1%)), Crepitus over the Shoulder, (15 (4.4%)), and Tachypnea, (21 (6.1%)). On auscultation of the pericardium, a rasping sound that coincides with each systole (Hamman's Sign) was reported in 38 (11.2%) of cases. Precipitating factors that could be associated with SPM were identified in 138 (40.7%) case reports. These included athletic activities, 45 (13.3%), Cough related to Valsalva, 44 (13%), Snorting, 19 (5.6%), Labor, 4 (1.2%), Sneezing, 4 (1.2%), severe retching /vomiting, 21 (6.2%), and Sex, 1 (0.3%) (Table 2).

**Table 2.** Precipitating factors that may have led to the development of Spontaneous Pneumomediastinum

Precipitating factor	Frequency
Athletics activities	45 (13.3)
Cough	44 (13)
Snorting	19 (5.6)
Labor	4 (1.2)
Sneezing	4 (1.2)
Sever reaching/vomiting	21 (6.2)
Sex	1 (1.6%)
Total	138 (40.7)

### Imaging studies

Chest radiography was used as the initial imaging study in 307 (90.6%) case reports. In 75 (22.1%) cases, PA and lateral views of the chest X-ray were used alone for the diagnosis of SPM. For the rest of 180 (53%) cases, an initial chest X-ray with subsequent CT-Scan of the chest was done. The remaining 32 (9.4%) cases were diagnosed using a CT-scan of the chest alone. Almost all cases, 315 (93%), with SPM has also associated subcutaneous emphysema. Other associated image findings were apical small Pneumothorax, 39 (11.5%), Pneumorrhachis (air inside the spinal canal) in 7 (2.06%), and Pneumoperitoneum in 3 (0.88%). The patients with pneumoperitoneum had generalized peritonitis due to tumor perforation and diverticulitis of the sigmoid colon.

Other diagnostic procedures

Additional diagnostic procedures to identify the possible cause of SPM were performed in 167 patients (49.3%) to identify the cause of the Pneumomediastinum (Airway/esophageal lesions). Gastrografin swallow to exclude esophageal perforation was done in 85 cases (25%). The bronchoscope was done for 30 (8.8%) patients. Only one tracheomalacia was reported. Findings were normal for the 52 (15.3%) patients who underwent esophagogastroduodenoscopy. Three case reports have used ultrasound as an additional diagnostic tool for SPM.

### Clinical course

Three hundred fifteen studied cases were hospitalized for a mean hospital length of 4.15 ± 1.93 days (range, 1-60 days). 24 patients were treated at OPD. Of these hospitalized patients, 167 (53%) were admitted primarily for SPM. In the remaining 148 patients (47%), hospitalization occurred for another additional reason too. These reasons included bronchial asthma, 57 (18%), Dermatomyositis/ Sclerosis/ Connective tissue disorder, 19 (6.03%), DKA, 8 (2.5%), interstitial lung disease, 13 (4.1%), Influenza-like syndrome, 4 (1.3%), cancer related complications, 12 (3.8%), COPD other than asthma, 7 (2.2%), Broncholithiasis, 5 (1.6%), Rabbis, 2 (0.56%) Tuberculosis, 3 (0.95%), anorexia Nervosa, 2 (0.56%), cystic lung disease, 2 (0.56%), Achalasia Cardia, 1 (0.31%), PCP/HIV, 2 (0.56%), Blebs, 1 (0.31%), MI, 1 (0.31%) and acute respiratory disorder in newborn due to 21-hydroxylase deficiency, 1 (0.31%) case (Table 3).

**Table 3:** Preexisting Diseases in 148 patients with Spontaneous

Asthma	57 (18)
Dermatomyositis	19 (6.03)
Cancer-related complications	12 (3.8)
DKA	8 (2.5)
COPD	7 (2.2)
Broncholithiasis	5 (1.6)
Tuberculosis	3 (0.95)
Cystic lung disease	2 (0.56
PCP/HIV	2 (0.56)
Influenza-like syndrome	4 (1.3)
Rabies	2 (0.56)
Anorexia Nervosa	2 (0.56)
Achalasia Cardia	1 (0.31)
Blebs	1 (0.31)
Myocardial infarction	1 (0.31)
21-hydroxylase deficiency	1 (0.31)
Total	148 (43.6)

DKA: Diabetic ketoacidosis, COPD: Chronic obstructive pulmonary disease, PCP/HIV: Pnumocystis carinii pneumonia/AIDS

Concomitant pneumothorax was identified in in 39 patients (11.5%). All of them except one had small apical pneumothorax that resolved with conservative management. One patient needed placement of bilateral chest tube, for bilateral spontaneous pneumothorax and Pneumomediastinum that complicate after an acute asthma attack. Subsequently, chemical pleurodesis with talcum powder was performed on his right lung, followed by his left lung on the next day.

Nearly 331 (97.6%) cases were treated conservatively with bed rest, antibiotics, bronchodilators steroids, high concentration oxygen and or careful watching. Antibiotics were administered in 112 patients (33%) out of concern for mediastinitis or sepsis. However, none of the study cases were documented to have sepsis or mediastinitis that is related to primary SPM during their hospital stay. Close to 19 (5.6%) of patients with connective tissue disorder received steroids treatment.

Two cases operated immediately for exploration of the abdomen for the possible perforation GI tract. Six patients had surgery after failed conservative treatment. Among those patients, one had perforated tracheal tumor for which tracheal resection and primary anastomosis were done, one had tracheomalacia and for whom repair was done. One underwent a video-assisted thoracoscopic surgery (VATS) procedure for a definite diagnosis of a sub-pleural bleb. The rest three had surgery with unremarkable operative findings.

There was no evidence of perforation in the upper esophagus and oropharynx. The retropharyngeal area was normal without any accumulation of fluid or purulent discharge.

Of the study cases, 302 (89.1%) had complete resolution of the SPM. The average duration needed for the complete resolution of Pneumomediastinum was 6.65 ± 11.8 days (Range 1-60 days). Nineteen patients (5.6%) died during their hospital stay: 2 as a result of complications related to rabies, 2 due to Dermatomyositis-associated interstitial lung disease, 2 following ARDS due to Swine Flu (H1N1) infection, and the rest because of MODS due to perforated diverticulitis of sigmoid colon, bleomycin-induced pulmonary interstitial disease given for germ cell tumor of testis, PCP related to HIV. An 88 years old lady was admitted for Primary SPM, while she was improving from her initial complaint (SPM), she suffered from CVA and subsequently died from hospital-acquired pneumonia, and the newborn died of respiratory failure after 6 h of delivery.

Patients were followed up for a median duration of 4 months (Range 0- 24 months). During follow up, Pneumomediastinum recurred in 9 patients; and no identifiable risk factor was found in them.

## Discussion

Lae ¨nnec, in 1819, was the first to describe Pneumomediastinum as a consequence of trauma [1–3]. Hamman, in 1939, published the first case series of SPM, as it is defined by the presence of free air in the mediastinum, not related to trauma or surgical procedures [4, 8, 9]. SPM is an uncommon entity with a prevalence that ranges between 0.001 and 0.01% [3]. The course of the disease is usually benign and often goes undiagnosed [1, 4, 5].

Mackline [1] has demonstrated that the pathophysiology of SPM is suggested to be an alveolar rapture, which results from high intra-alveolar pressure, low perivascular pressure, or both. The leaking air will ascend along with the pulmonary interstitial space towards the subcutaneous space of the mediastinum, neck, face, chest wall, abdomen, and even the limbs resulting in subcutaneous emphysema [1–3, 6, 9].

Precipitating factors that could be responsible for the cause of SPM were identified in 34% of Iyer *et al.* [4] report. A similar finding (40.7%), were also found in our study. Among those who reported triggers SPM are snorting, Physical exercise, labor, Diabetic ketoacidosis (DKA), cough, and severe retching/vomiting. SPM has been associated to be related to a variety of lung diseases that include asthma, COPD, emphysema, interstitial lung diseases, and bronchiectasis [1, 3].

These diseases were also reported in 43.6% of our cohort of case reports.

SPM is frequently seen in young males with an average age of between 20–30 years. (2–12) Inconsistent with many other authors, (1–8) the most common presenting symptoms reported were, dyspnea, chest pain, cough, neck swelling, and cervical pain. In about 10% of conditions, SPM could be incidentally found during routine investigations done for other problems. [10] Similarly, 8.7% of reported cases in this study were found incidentally. Ryoo [3] reported that the average time from onset to the first visit to the hospital was 1 day. Similarly, in our study, 58% of the cases had < 2 days of symptoms.

In our study, 315 (93%) patients with SPM have also associated subcutaneous emphysema detected in imaging studies. However, only 45.4% were detected with a physical examination. Subcutaneous emphysema was seen in 68% of patients reported by Takada K *et al.* [10] Different authors reported a similar finding with a range of 40% to 100% [1–10]. Some authors consider the presence of subcutaneous emphysema as a good sign because the air leaking into the subcutaneous tissue prevents an increase in intra-mediastinum pressure. A well-known auscultatory finding of SPM, Hamman's sign (rasping sound heard during each heartbeat) was reported in 11.2% of this study. Other authors also describe such findings as uncommon [10–12].

The initial imaging study done in suspected cases of SPM is chest X-ray. The sensitivity of this study exceeds 90% [4]. The imaging signs seen include thymic sail sign (an elevated thymus by air), Pneumopericardium (air anterior to pericardium), the air surrounding pulmonary artery, air adjacent to major branches of the aorta, and tubular artery sign (mediastinal air outlines on the medial side with the aerated lung marginates the lateral side) [6].

In a study done by Kaneki *et al.* [13], 30% of mild SPM was not detected by a simple chest X-ray and a CT scan of the lung was additionally used for the diagnosis. Similarly, we reported 53% of cases had an initial chest x-ray with subsequent CT-Scan. As reported in the above initial case report, aside from being more accurate in detecting small amounts of air in the mediastinum, a CT scan of the chest can occasionally reveal other findings that may provide insights into the underlying cause or associated disease [11, 13].

Additional diagnostic test like gastografine swallow, bronchoscopy, and upper GI endoscopy has a limited role in the evaluation of patients with SPM. In our study, 167 (49.3%) patients had a bronchoscopy, gastografine swallow, or upper GI endoscopy. None of these procedures yielded relevant findings. Our findings on this issue are consistent with those of previously published reports [1–5].

The frequency of concomitant pneumothorax (11.5%) as seen in our study was similar to other studies that report a frequency of 6–11% [1–9]. Other studies done by Iyer et al [4] reported a higher frequency (32%). Their higher rate of pneumothorax is probably due to a higher prevalence of preexisting lung diseases, and a higher mean age of the study participants. Other associated image findings reported in our report were Pneumorrhachis (air inside the spinal canal) and Pneumoperitoneum. Both are reported in 7 (2.06%) and 3 (0.88%) cases respectively. Reported patients with pneumoperitoneum had generalized peritonitis due to perforation of the sigmoid colon due to tumor perforation and diverticulitis. Pneumorrhachis and pneumoperitoneum were even rare, with only a few cases reported in the literature [7, 14, 15]. Rarely, during SPM, air from the posterior mediastinum may go through the neural foramina into the epidural space to cause pneumorrhachis [7]. Pneumoperitoneum due to bowel perforation could be found along with pneumomediastinum. This happens because the air from extra-peritoneal space escape could diffuse through paravertebral retroperitoneal tissue via the diaphragmatic hiatus into the mediastinum [15].

According to our result, 148 (43.6%) cases have been associated with other diseases like bronchial asthma, Dermatomyositis, DKA, Influenza-like syndrome, cancer-related complications, COPD other than asthma, rabies, tuberculosis, anorexia nervosa, achalasia cardia, Pnumocystis carinii pneumonia/AIDS (PCP/HIV), Blebs, MI, and acute respiratory disorder in newborn due to 21-hydroxylase deficiency. In the case reported here, the clinical history, CT scan, and other laboratory findings ruled out those diagnoses.

The treatment for SPM is controversial. Most studied articles, 331 (97.6%), used conservative treatment-including bed rest, analgesia, a bronchodilator, steroids, and antibiotics [2]. On the other hand, some centers have recommended that invasive tests and antimicrobial agents are used sparingly and dietary restrictions are avoided, given that all of these increase the mean length of hospital stay [4]. Besides, unfamiliarity with the entity can lead to unnecessary diagnostic tests and inappropriate treatment [2].

The complications of SPM vary according to the etiology or the triggering factor. In some cases, delayed diagnosis and failure to detect the primary cause of SPM can lead to life-threatening complications like esophageal rupture, mediastinitis, peritonitis, and tension pneumothorax, among others [1, 4, 5]. In our study, 19 patients (5.6%) died during their hospital stay. All deaths are related either due to interstitial lung disease, ARDS, or MODS because of the underlining disease condition not related to SPM. Recurrence is rare, reported in only three cases of our cohort, long-term follow-up being therefore unnecessary.

## Conclusion

In the present study, we have shown that SPM is a rare disease with < 200 full-text English articles published worldwide. The true prevalence, however, could be higher if articles written in other languages are included. The study was entirely based on case reports and small case series, which make the level of evidence presented below. However, our data and many retrospective cohort studies suggest that SPM is a rare disorder, usually benign and self-limited that occurs primarily in young male patients, associated with a diverse clinical feature with characteristic Chest X-ray and CT scan findings. In the absence of a concomitant pneumothorax/pneumoperitoneum/pneumorrhachis or severe underlying illness requiring inpatient treatment, patients with SPM could be treated on an outpatient basis. The clinical course of patients with SPM is influenced more by the severity of the underlying disorder (e.g. COPD, Severe asthma, Dermatomyositis, DKA, Influenza-like syndrome, cancer-related complications) than by the SPM itself. Diagnostic procedures performed to identify any underlying anatomic cause (e.g. esophageal or tracheobronchial rupture) have a low yield and are unnecessary in the majority of cases.

## Confirmation

We confirm that the content of the manuscript has not been published, or submitted for publication elsewhere.

## Data Availability

A soft copy of all data used for this article is available with the corresponding author
